# Potential anti-osteoporotic and anti-inflammatory potential of plant derived extracts and column semi-purified fractions of Epimedium brevicornum

**DOI:** 10.3389/fcell.2026.1938049

**Published:** 2026-10-08

**Authors:** Nan Ding, Baohui Wang

**Affiliations:** 1 Department of Traditional Chinese Medicine Rehabilitation, Honghui Hospital, Xi’an Jiaotong University, Xi'an, China; 2 Traditional Chinese Medicine Pain Department, TCM Specialty Diagnosis and Treatment Center, Honghui Hospital, Xi’an Jiaotong University, Xi'an, China

**Keywords:** bone resorption, Epimedium brevicornum, natural products, osteoporosis, TRAP inhibition

## Abstract

**Background:**

Osteoporosis is characterized by excessive bone resorption and impaired bone remodeling, often accompanied by inflammatory processes that exacerbate skeletal deterioration. Natural products with multi-target potential have gained attention as alternative therapeutic strategies. The present study aimed to investigate the *in-vitro* anti-osteoporotic and anti-inflammatory potential of extracts obtained from different parts of Epimedium brevicornum, including leaves, stem, and roots, as well as isolated column semi-purified fractions (E-B-1 C, E-B-2 C, E-B-3 C, and E-B-4 C) derived from the leaf extract.

**Methods:**

Crude methanolic extracts and leaf-derived semi-purified fractions were prepared using column chromatography and evaluated for inhibitory activity against key enzymes involved in bone resorption and remodeling, including tartrate-resistant acid phosphatase (TRAP), cathepsin K, matrix metalloproteinase-9 (MMP-9), and matrix metalloproteinase-13 (MMP-13). Anti-inflammatory activity was assessed through inhibition of cyclooxygenase-2 (COX-2) and 5-lipoxygenase (5-LOX). Enzyme inhibition assays were conducted at varying concentrations to determine percentage inhibition and IC_50_.

**Results:**

All tested extracts and fractions demonstrated concentration-dependent inhibition across the evaluated targets. Among the semi-purified fractions, E-B-2 C and E-B-3 C generally showed stronger inhibitory activities against bone resorption-related enzymes, whereas E-B-1 C exhibited the strongest inhibition of 5-LOX. The IC_50_ values ranged from 95.22 to 462.37 μg/mL across the six enzymatic targets. Overall, the semi-purified fractions showed greater inhibitory potency than the crude extracts.

**Conclusion:**

The findings suggest that Epimedium brevicornum may serve as a promising natural source of bioactive compounds with dual anti-osteoporotic and anti-inflammatory properties. Further investigations focusing on isolation, structural characterization, and *in-vivo* validation are warranted to confirm therapeutic potential.

## 1 Introduction

Osteoporosis is a systemic skeletal disorder characterized by decreased bone mass and deterioration of bone microarchitecture, ultimately leading to enhanced bone fragility and increased susceptibility to fractures (Yong and Logan, 2021). It is widely recognized as one of the most prevalent metabolic bone diseases worldwide (Walker and Shane, 2023). With the progressive aging of the global population, the incidence and socioeconomic burden of osteoporosis continue to rise (Kanis et al., 2012). Epidemiological data indicate that millions of individuals suffer from osteoporotic fractures annually, particularly hip, vertebral, and wrist fractures, which are associated with increased morbidity, mortality, and healthcare expenditure (Walker and Shane, 2023; Kanis et al., 2012). Postmenopausal women are especially vulnerable due to estrogen deficiency, while elderly men are also at significant risk due to age-related hormonal changes and impaired bone remodeling (Armas and Recker, 2012). The growing prevalence of osteoporosis has therefore become a major public health concern across both developed and developing countries.

Bone homeostasis is maintained through a dynamic and tightly regulated process known as bone remodeling, which involves a balance between bone formation by osteoblasts and bone resorption by osteoclasts (Raggatt and Partridge, 2010). Disruption of this balance, particularly excessive osteoclastic activity, plays a central role in the pathogenesis of osteoporosis (Udagawa et al., 2020). Osteoclasts resorb bone matrix through the coordinated action of several key enzymes. Tartrate-resistant acid phosphatase (TRAP) is widely regarded as a biochemical marker of osteoclast differentiation and activity (Habermann et al., 2007). Cathepsin K is a lysosomal cysteine protease highly expressed in osteoclasts and is responsible for the degradation of type I collagen, the major organic component of bone matrix (Ren et al., 2015; Yang et al., 2020). In addition, matrix metalloproteinases (MMPs), particularly MMP-9 and MMP-13, contribute to extracellular matrix remodeling and collagen breakdown during bone resorption (Mustafa et al., 2025). Elevated activity of these enzymes has been associated with increased bone loss and structural deterioration (Ståhle-Bäckdahl et al., 1997). Therefore, inhibition of bone-resorptive enzymes represents a promising therapeutic approach for preventing excessive bone degradation.

A variety of pharmacological agents are currently available for the management of osteoporosis, including bisphosphonates, selective estrogen receptor modulators, parathyroid hormone analogs, monoclonal antibodies targeting RANKL, and sclerostin inhibitors (Silverstein et al., 2024). Although these medications have demonstrated efficacy in reducing fracture risk and improving bone mineral density, long-term therapy is often associated with adverse effects (Borrelli et al., 2014). Reported complications include gastrointestinal disturbances, atypical femoral fractures, osteonecrosis of the jaw, increased cardiovascular risk, and rapid bone loss upon drug discontinuation (Jain, 2024). Furthermore, patient compliance remains suboptimal due to dosing regimens and safety concerns. These limitations highlight the need for safer and alternative therapeutic strategies that can effectively modulate bone remodeling with fewer side effects. In addition to bone-resorptive enzymes, inflammation has emerged as an important contributing factor in osteoporosis (Yu and Wang, 2022). Chronic low-grade inflammation is known to enhance osteoclast differentiation and activity through various mediators. Pro-inflammatory cytokines can stimulate the expression of enzymes involved in bone degradation and accelerate bone turnover (Raisz, 2005). Cyclooxygenase-2 (COX-2) and 5-lipoxygenase (5-LOX) are key enzymes involved in inflammatory signaling pathways, leading to the production of prostaglandins and leukotrienes. Increased expression of COX-2 and 5-LOX has been associated with bone metabolic imbalance and inflammatory bone loss (Raisz, 2005). Therefore, compounds exhibiting both anti-resorptive and anti-inflammatory properties may offer dual therapeutic benefits in the management of osteoporosis.

Natural products derived from medicinal plants have gained considerable attention in recent decades due to their multi-target potential and relatively favorable safety profiles (Arena et al., 2022). Historically, plant-based remedies have been widely used in traditional medicine systems for the treatment of various chronic disorders. It has been reported that a substantial proportion of modern pharmaceuticals are either directly or indirectly derived from plant sources. The increasing interest in phytochemicals as potential therapeutic agents reflects their structural diversity and biological versatility. In the context of bone health, several plant-derived compounds have demonstrated inhibitory effects on osteoclast differentiation and enzyme activity in experimental models (Ahirwar et al., 2026).

*Epimedium brevicornum*, commonly known as Horny Goat Weed, is a traditional medicinal herb widely used in East Asian medicine. It belongs to the family Berberidaceae and has been traditionally prescribed for conditions related to bone weakness, aging, and general vitality. Phytochemical investigations of *Epimedium* species have revealed the presence of diverse bioactive constituents, including flavonoids, prenylated flavonol glycosides, phytosterols, and phenolic derivatives (Chen et al., 2024). Major compounds reported in *E. brevicornum* include icariin, icariside A7, anhydroicaritin, kaempferol, quercetin, luteolin, chryseriol, sitosterol, and various YinYanghuo derivatives (Chen et al., 2023). These constituents are believed to contribute to the pharmacological properties of the plant. Although previous studies have explored isolated compounds from *Epimedium* species, limited investigations have comparatively evaluated crude extracts and column semi-purified fractions for their enzyme inhibitory activity against bone-resorption-related targets. Fractionation of crude extracts may enrich specific bioactive constituents and enhance biological activity. Therefore, systematic evaluation of different plant parts and their semi-purified fractions may provide insight into their therapeutic potential.

In light of the above considerations, the present study was designed to investigate the *in-vitro* anti-osteoporotic and anti-inflammatory potential of extracts obtained from leaves, stem, and roots of *E. brevicornum*, along with isolated column semi-purified fractions. The inhibitory activity against TRAP, cathepsin K, MMP-9, MMP-13, COX-2, and 5-LOX was evaluated to assess the potential of this plant as a natural source of bioactive compounds for the management of osteoporosis. These six enzymes were selected as a complementary target panel representing three closely related processes involved in osteoporosis: osteoclast-mediated bone resorption (TRAP and cathepsin K), extracellular matrix degradation (MMP-9 and MMP-13), and inflammation (COX-2 and 5-LOX).

## 2 Materials and methods

### 2.1 Plant collection

Fresh samples of *E. brevicornum* were collected in May 2025 from Shaanxi Province, China. The plant material was taxonomically authenticated by a qualified botanist at Honghui Hospital affiliated institution. A voucher specimen (EB-2025-01) was deposited in the institutional herbarium for future reference. The collected plant materials were washed, shade-dried at 25 °C ± 2 °C, and pulverized into coarse powder for extraction.

### 2.2 Extraction and preparation of subfractions

The powdered leaves, stems, and roots (2.2 kg each) were separately macerated in methanol (analytical grade) at a ratio of 1:5 (w/v) for 72 h at room temperature with intermittent shaking. The extracts were filtered through Whatman No.1 filter paper and concentrated under reduced pressure at 40 °C using a rotary evaporator (Rotavapor® R-300, BUCHI Corporation, USA) to obtain crude methanolic extracts. Thin Layer Chromatography (TLC) profiling was performed on silica gel plates using chloroform:methanol (8:2) as the mobile phase to evaluate chemical diversity. To ensure consistency, all batches were prepared using the same plant-to-solvent ratio, extraction conditions, and concentration procedure. Batch quality was verified by TLC profiling under identical chromatographic conditions, and batches showing comparable chromatographic profiles were used for subsequent assays. The methanolic leaf extract, which showed comparatively higher constituent complexity based on TLC profiling, was subjected to silica gel column chromatography (70–230 mesh). Elution was carried out using chloroform:methanol gradient solvent systems, yielding four semi-purified fractions designated as E-B-1 C (7:3), E-B-2 C (7.5:3.5), E-B-3 C (6:4), and E-B-4 C (6.5:3.5). Fractions were pooled based on TLC similarity, concentrated, dried, and stored at −20 °C until further biological evaluation. All samples were dissolved in dimethyl sulfoxide (DMSO) prior to use, with final DMSO concentration maintained below 1%.

### 2.3 *In vitro* anti-osteoporotic activity

The inhibitory activity of crude extracts and semi-purified fractions against bone-resorption-related enzymes was evaluated at concentrations ranging from 62.5 to 1,000 μg/mL. For all enzyme assays, percentage inhibition was calculated using the following equation:

$$Inhibition\;\left(\%\right)=\frac{AC-AS}{AC}\times 100$$

where AC represents the absorbance (or fluorescence intensity) of the control reaction without test sample, and AS represents the absorbance (or fluorescence intensity) in the presence of the test sample. The IC_50_ values (concentration required to inhibit 50% of enzyme activity) were determined by nonlinear regression analysis using GraphPad Prism seven software.

#### 2.3.1 TRAP inhibitory assay

Tartrate-resistant acid phosphatase (TRAP) inhibitory activity was determined using a p-nitrophenyl phosphate (pNPP) substrate-based colorimetric assay. The reaction mixture (final volume 1 mL) consisted of 50 mM sodium acetate buffer (pH 5.5) containing 10 mM sodium tartrate, TRAP enzyme solution (1 U/mL), and test sample (62.5–1,000 μg/mL). After pre-incubation at 37 °C for 20 min, 5 mM pNPP substrate (Sigma-Aldrich, St. Louis, MO, USA; Cat. No. P4744) was added and the reaction was further incubated at 37 °C for 30 min. The reaction was terminated by addition of 0.1 M NaOH, and absorbance was measured at 405 nm using a UV–Vis spectrophotometer. Sodium orthovanadate was used as a reference inhibitor. Percentage inhibition was calculated using the equation provided above.

#### 2.3.2 Cathepsin K inhibitory assay

Cathepsin K inhibitory activity was assessed using a Cathepsin K Inhibitor Screening Assay Kit (Abcam, Cambridge, UK; Cat. No. ab157403) in 50 mM sodium acetate buffer (pH 5.5) containing 5 mM DTT. The enzyme solution (0.5 U/mL) was incubated with test samples (62.5–1,000 μg/mL) at 37 °C for 20 min prior to substrate addition. The reaction was initiated by adding 200 μM Z-Gly-Pro-Arg-pNA substrate and incubated for 30 min at 37 °C. Absorbance was recorded at 405 nm. E−64 was used as positive control. Percentage inhibition and IC_50_ values were calculated according to the equation stated above.

### 2.4 MMP-9 and MMP-13 inhibitory assay

The inhibitory activity against MMP-9 and MMP-13 was determined using fluorometric assays with MMP-9 and MMP-13 Inhibitor Screening Assay Kits (Abcam, Cambridge, UK; Cat. Nos. ab139449 and ab139451, respectively). The reaction mixture contained 50 mM Tris-HCl buffer (pH 7.5), 10 mM CaCl_2_, 0.05% Brij-35, enzyme solution (1 U/mL), and test sample (62.5–1,000 μg/mL). After pre-incubation at 37 °C for 15 min, fluorogenic peptide substrate (10 μM) was added and fluorescence was monitored at excitation 320 nm and emission 405 nm for 30 min. The rate of fluorescence increase was calculated, and percentage inhibition was determined using the formula:

$$Inhibition\;\left(\%\right)=\frac{VC-VS}{VC}\times 100$$

where VC is the reaction velocity (fluorescence change per minute) of the control and VS is the reaction velocity in the presence of test sample. IC_50_ values were calculated using nonlinear regression analysis.

### 2.5 *In vitro* anti-inflammatory activity

#### 2.5.1 COX-2 inhibitory assay

COX-2 inhibitory activity was evaluated using a colorimetric assay with a COX Colorimetric Inhibitor Screening Assay Kit (Cayman Chemical, Ann Arbor, MI, USA; Cat. No. 701050). The reaction mixture contained 0.1 M Tris-HCl buffer (pH 8.0), hematin (1 mM), TMPD (0.24 mM), glutathione (0.9 mM), COX-2 enzyme (300 U/mL), and test sample (62.5–1,000 μg/mL). After incubation at 25 °C for 5 min, the reaction was initiated by addition of 30 mM arachidonic acid. Absorbance was measured at 540 nm. Celecoxib was used as reference drug. Percentage inhibition was calculated as:

$$Inhibition\;\left(\%\right)=\frac{AC-AS}{AC}\times 100$$

IC_50_ values were obtained by plotting inhibition percentage against log concentration using nonlinear regression.

#### 2.5.2 5-LOX inhibitory assay

For 5-LOX inhibitory activity, the reaction mixture consisted of 50 mM phosphate buffer (pH 6.3), 5-LOX enzyme (lipoxidase from Glycine max, Sigma-Aldrich, St. Louis, MO, USA; Cat. No. L6632) (10,000 U/mL), and test sample (62.5–1,000 μg/mL). After incubation at 25 °C for 5 min, the reaction was initiated by addition of 250 μM linoleic acid substrate. Absorbance was recorded at 234 nm. Percentage inhibition was calculated using:

$$Inhibition\;\left(\%\right)=\frac{AC-AS}{AC}\times 100$$

### 2.6 Phytochemical profiling

The crude methanolic extract of *E. brevicornum* was subjected to LC–MS analysis for phytochemical profiling. The analysis revealed the presence of flavonoids, prenylated flavonol glycosides, phytosterols, and phenolic derivatives, including icariin, icariside A7, anhydroicaritin, kaempferol, quercetin, luteolin, chryseriol, sitosterol, and YinYanghuo derivatives. Identified constituents are summarized in Table 1.

**TABLE 1:** Anti-inflammatory enzyme inhibitory activity of Epimedium extracts and fractions.

Samples	Concentration (µg/mL)	COX-2 inhibition (mean ± SEM)	IC50 (µg/mL)	5-LOX inhibition (mean ± SEM)	IC50 (µg/mL)
E-B-1 C	1,000	65.4 ± 0.5***	319.06	73.66 ± 0.54***	119.25
	500	55.59 ± 0.3***	​	62.61 ± 0.26***	​
	250	45.78 ± 0.3***	​	51.56 ± 0.21***	​
	125	32.7 ± 0.39***	​	36.83 ± 0.48***	​
	62.5	19.62 ± 0.27***	​	22.1 ± 0.25***	​
E-B-2 C	1,000	67.05 ± 0.24***	110.11	73.12 ± 0.24***	209.68
	500	56.99 ± 0.47***	​	62.16 ± 0.22***	​
	250	46.93 ± 0.25***	​	51.19 ± 0.42***	​
	125	33.52 ± 0.43***	​	36.56 ± 0.25***	​
	62.5	20.11 ± 0.27***	​	21.94 ± 0.31***	​
E-B-3 C	1,000	69.46 ± 0.21***	198.25	67.02 ± 0.47***	138.0
	500	59.04 ± 0.35***	​	56.97 ± 0.22***	​
	250	48.63 ± 0.5***	​	46.92 ± 0.3***	​
	125	34.73 ± 0.3***	​	33.51 ± 0.21***	​
	62.5	20.84 ± 0.48***	​	20.11 ± 0.37***	​
E-B-4 C	1,000	71.23 ± 0.46***	171.22	70.07 ± 0.23***	219.08
	500	60.55 ± 0.24***	​	59.56 ± 0.36***	​
	250	49.86 ± 0.19***	​	49.05 ± 0.24***	​
	125	35.62 ± 0.23***	​	35.03 ± 0.55***	​
	62.5	21.37 ± 0.54***	​	21.02 ± 0.52***	​
Leaf	1,000	65.06 ± 0.3***	213.76	68.63 ± 0.3***	232.86
	500	55.3 ± 0.42***	​	58.34 ± 0.22***	​
	250	45.54 ± 0.34***	​	48.04 ± 0.35***	​
	125	32.53 ± 0.54***	​	34.32 ± 0.47***	​
	62.5	19.52 ± 0.45***	​	20.59 ± 0.32***	​
Stem	1,000	73.25 ± 0.55***	120.89	65.56 ± 0.2***	337.66
	500	62.26 ± 0.3***	​	55.73 ± 0.36***	​
	250	51.27 ± 0.24***	​	45.89 ± 0.4***	​
	125	36.62 ± 0.46***	​	32.78 ± 0.51***	​
	62.5	21.97 ± 0.46***	​	19.67 ± 0.39***	​
Root	1,000	73.04 ± 0.5***	151.12	65.78 ± 0.3***	273.91
	500	62.08 ± 0.55***	​	55.92 ± 0.25***	​
	250	51.13 ± 0.51***	​	46.05 ± 0.33***	​
	125	36.52 ± 0.24***	​	32.89 ± 0.22***	​
	62.5	21.91 ± 0.39***	​	19.74 ± 0.38***	​

*** indicates p < 0.001 versus the positive control.

### 2.7 Statistical analysis

All experiments were performed in triplicate (n = 3), and results were expressed as mean ± SEM. Statistical significance was evaluated using two-way analysis of variance (ANOVA) followed by Bonferroni *post hoc* test. A p-value <0.05 was considered statistically significant. IC_50_ values were calculated using nonlinear regression analysis in GraphPad Prism seven software (GraphPad Software, USA).

## 3 Results

### 3.1 *In vitro* anti-osteoporotic potential

The crude extracts obtained from leaves, stems, and roots of *E. brevicornum*, along with their isolated column semi-purified fractions derived from the methanolic leaf extract (E-B-1 C, E-B-2 C, E-B-3 C, and E-B-4 C), were evaluated for inhibitory activity against TRAP and cathepsin K enzymes. All tested samples demonstrated concentration-dependent inhibition within the range of 62.5–1,000 μg/mL. At the highest tested concentration (1,000 μg/mL), E-B-2 C exhibited the strongest TRAP inhibition (71.84% ± 0.42%), followed by E-B-3 C (67.92% ± 0.37%) and leaf extract (69.15% ± 0.28%). The root extract showed comparatively lower inhibition (55.33% ± 0.31%). The calculated IC_50_ values ranged from 118.45 to 462.37 μg/mL, with E-B-2 C demonstrating the lowest IC_50_ value among tested samples.

Similarly, Cathepsin K inhibition was highest for E-B-3 C (73.21% ± 0.39%) at 1,000 μg/mL, followed by E-B-2 C (70.65% ± 0.41%) and leaf extract (72.48% ± 0.35%). The root extract showed moderate activity (58.14% ± 0.44%). IC_50_ ranged from 102.67 to 389.55 μg/mL (Figures 1, 2). The detailed percentage inhibition at each concentration and corresponding IC_50_ values are presented in Table 2.

**FIGURE 1:**
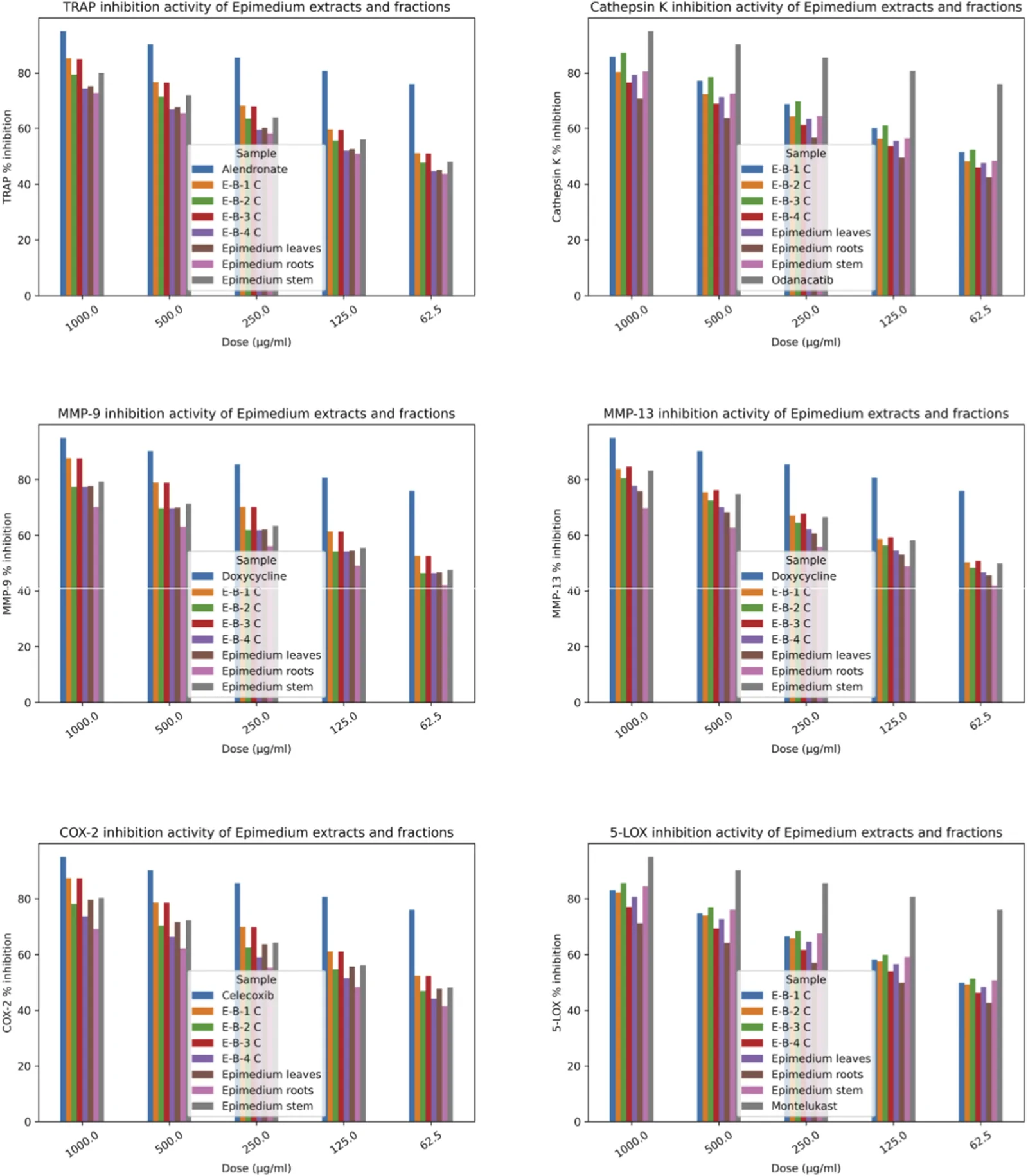
Percent inhibition of six key enzymes involved in bone resorption and inflammatory pathways by crude extracts and column semi-purified fractions of Epimedium brevicornum. The inhibitory activity of crude extracts and column semi-purified fractions of Epimedium brevicornum (E-B-1 C, E-B-2 C, E-B-3 C, and E-B-4 C), together with different plant parts (leaf, stem, and root extracts), was evaluated against six enzymes associated with bone metabolism and inflammation, including tartrate-resistant acid phosphatase (TRAP), cathepsin K, matrix metalloproteinase-9 (MMP-9), matrix metalloproteinase-13 (MMP-13), cyclooxygenase-2 (COX-2), and 5-lipoxygenase (5-LOX), at concentrations of 1,000, 500, 250, 125, and 62.5 μg/mL. Alendronate, odanacatib, doxycycline, celecoxib, and montelukast were used as positive controls for the respective enzyme assays. Data are presented as mean ± standard error of the mean (S.E.M.), n = 3. Statistical significance was determined using two-way ANOVA followed by Bonferroni *post hoc* test. Values significantly different from the positive control are indicated as *p < 0.05, **p < 0.01, and ***p < 0.001.

**FIGURE 2:**
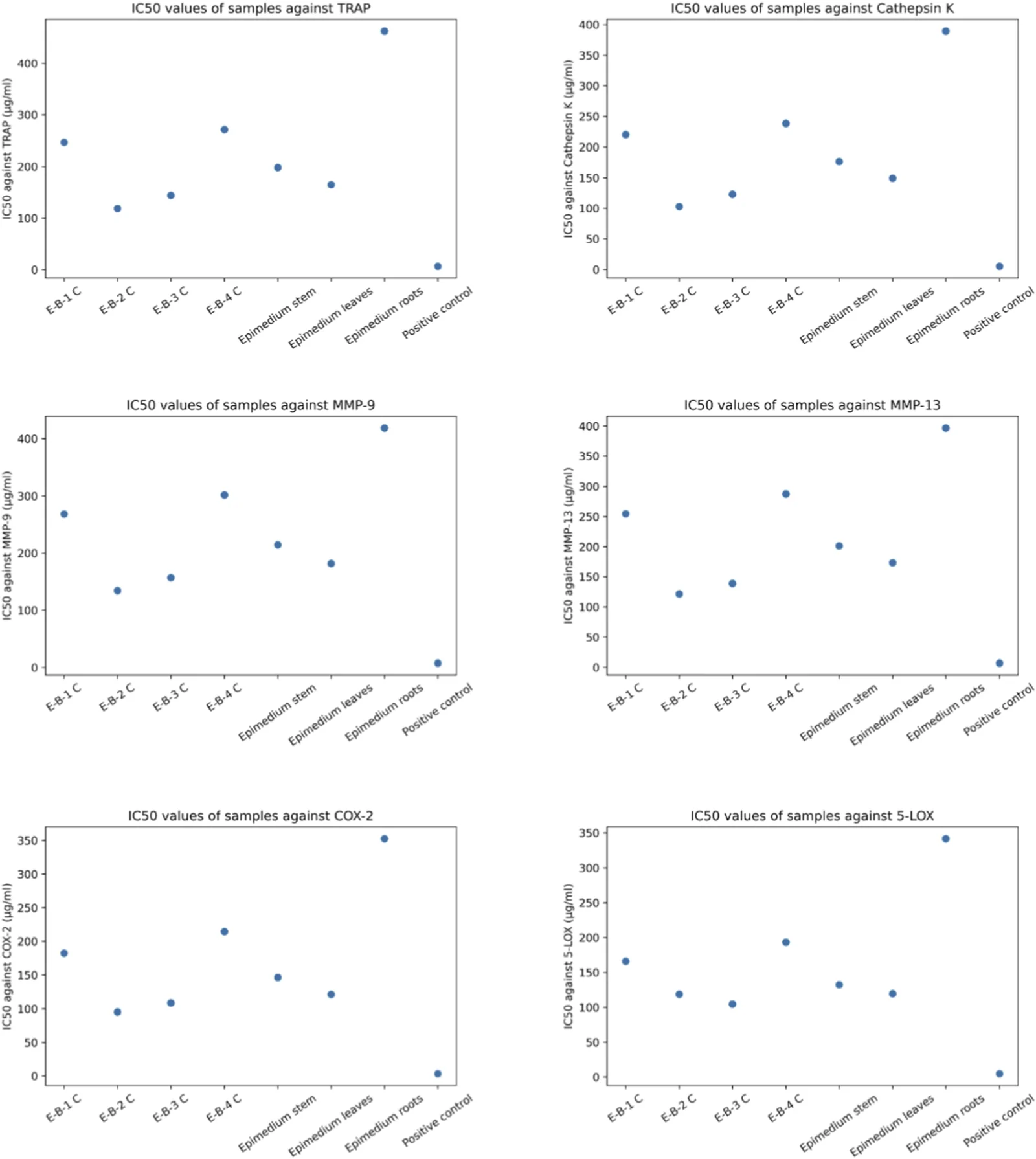
IC_50_ of crude extracts and column semi-purified fractions of Epimedium brevicornum against six enzymes related to bone resorption and inflammatory responses. The half-maximal inhibitory concentration (IC_50_) values of Epimedium brevicornum extracts, column semi-purified fractions (E-B-1 C, E-B-2 C, E-B-3 C, and E-B-4 C), and plant parts (leaf, stem, and root extracts) were determined against six enzymatic targets, including TRAP, cathepsin K, MMP-9, MMP-13, COX-2, and 5-LOX. The IC_50_ values were calculated from concentration–response curves using GraphPad Prism software. Data represent mean ± S.E.M. (n = 3). Lower IC_50_ values indicate stronger inhibitory activity against the corresponding enzyme. Data are presented as mean ± SEM (n = 3). Statistical significance was determined using two-way ANOVA followed by Bonferroni *post hoc* test. Exact p-values are indicated in the figure.

**TABLE 2:** Anti-osteoporotic enzyme inhibitory activity of Epimedium extracts and fractions.

Samples	Concentration (µg/mL)	TRAP inhibition (mean ± SEM)	IC50 (µg/mL)	Cathepsin K inhibition (mean ± SEM)	IC50 (µg/mL)
E-B-1 C	1,000	73.95 ± 0.48***	302.84	67.73 ± 0.53***	180.17
	500	62.86 ± 0.25***	​	57.57 ± 0.23***	​
	250	51.77 ± 0.49***	​	47.41 ± 0.24***	​
	125	36.98 ± 0.24***	​	33.86 ± 0.41***	​
	62.5	22.19 ± 0.35***	​	20.32 ± 0.37***	​
E-B-2 C	1,000	67.87 ± 0.21***	339.21	68.0 ± 0.22***	266.13
	500	57.69 ± 0.22***	​	57.8 ± 0.29***	​
	250	47.51 ± 0.49***	​	47.6 ± 0.39***	​
	125	33.93 ± 0.27***	​	34.0 ± 0.36***	​
	62.5	20.36 ± 0.52***	​	20.4 ± 0.48***	​
E-B-3 C	1,000	70.67 ± 0.21***	144.75	65.92 ± 0.22***	246.62
	500	60.07 ± 0.42***	​	56.03 ± 0.51***	​
	250	49.47 ± 0.38***	​	46.14 ± 0.54***	​
	125	35.34 ± 0.54***	​	32.96 ± 0.29***	​
	62.5	21.2 ± 0.44***	​	19.78 ± 0.47***	​
E-B-4 C	1,000	67.26 ± 0.27***	334.73	69.67 ± 0.28***	207.52
	500	57.17 ± 0.2***	​	59.22 ± 0.38***	​
	250	47.08 ± 0.21***	​	48.77 ± 0.49***	​
	125	33.63 ± 0.37***	​	34.83 ± 0.47***	​
	62.5	20.18 ± 0.53***	​	20.9 ± 0.3***	​
Leaf	1,000	72.28 ± 0.5***	144.64	70.55 ± 0.45***	184.7
	500	61.43 ± 0.19***	​	59.97 ± 0.39***	​
	250	50.59 ± 0.53***	​	49.38 ± 0.51***	​
	125	36.14 ± 0.47***	​	35.27 ± 0.37***	​
	62.5	21.68 ± 0.27***	​	21.16 ± 0.36***	​
Stem	1,000	65.64 ± 0.19***	124.3	72.75 ± 0.29***	174.25
	500	55.79 ± 0.2***	​	61.84 ± 0.21***	​
	250	45.95 ± 0.52***	​	50.92 ± 0.42***	​
	125	32.82 ± 0.29***	​	36.37 ± 0.3***	​
	62.5	19.69 ± 0.48***	​	21.82 ± 0.29***	​
Root	1,000	70.43 ± 0.51***	152.59	69.55 ± 0.26***	292.66
	500	59.87 ± 0.23***	​	59.12 ± 0.45***	​
	250	49.3 ± 0.47***	​	48.69 ± 0.29***	​
	125	35.22 ± 0.51***	​	34.78 ± 0.25***	​
	62.5	21.13 ± 0.48***	​	20.87 ± 0.38***	​

*** indicates p < 0.001 versus the positive control.

### 3.2 *In-vitro* anti-bone matrix degrading enzyme activity

The inhibitory activity of extracts and semi-purified fractions against MMP-9 and MMP-13 was evaluated using fluorometric assays. All samples showed concentration-dependent suppression of enzyme activity. At 1,000 μg/mL, E-B-3 C demonstrated the highest inhibition against MMP-9 (68.73% ± 0.36%), followed by E-B-2 C (65.49% ± 0.30%) and leaf extract (66.87% ± 0.42%). Root extract exhibited comparatively lower inhibition (53.21% ± 0.27%). The IC_50_ values for MMP-9 inhibition ranged from 134.29 to 418.76 μg/mL. For MMP-13 inhibition, E-B-2 C exhibited the strongest effect (70.88% ± 0.33%) at 1,000 μg/mL, followed by E-B-3 C (69.14% ± 0.38%) and leaf extract (71.02% ± 0.29%). Root extract demonstrated moderate inhibition (56.49% ± 0.35%). IC_50_ ranged between 121.54 and 396.83 μg/mL (Figures 1, 2). Complete data sets are summarized in Table 3.

**TABLE 3:** Matrix degradation enzyme inhibitory activity of Epimedium extracts and fractions.

Samples	Concentration (µg/mL)	MMP-9 inhibition (mean ± SEM)	IC50 (µg/mL)	MMP-13 inhibition (mean ± SEM)	IC50 (µg/mL)
E-B-1 C	1,000	69.53 ± 0.54***	162.55	74.98 ± 0.21***	216.04
	500	59.1 ± 0.54***	​	63.73 ± 0.24***	​
	250	48.67 ± 0.47***	​	52.48 ± 0.35***	​
	125	34.77 ± 0.53***	​	37.49 ± 0.49***	​
	62.5	20.86 ± 0.48***	​	22.49 ± 0.27***	​
E-B-2 C	1,000	74.68 ± 0.23***	333.82	71.52 ± 0.36***	336.38
	500	63.48 ± 0.33***	​	60.79 ± 0.44***	​
	250	52.27 ± 0.37***	​	50.06 ± 0.31***	​
	125	37.34 ± 0.34***	​	35.76 ± 0.39***	​
	62.5	22.4 ± 0.29***	​	21.46 ± 0.55***	​
E-B-3 C	1,000	71.76 ± 0.21***	173.66	71.58 ± 0.4***	121.49
	500	61.0 ± 0.27***	​	60.84 ± 0.55***	​
	250	50.23 ± 0.19***	​	50.1 ± 0.48***	​
	125	35.88 ± 0.36***	​	35.79 ± 0.44***	​
	62.5	21.53 ± 0.25***	​	21.47 ± 0.23***	​
E-B-4 C	1,000	69.77 ± 0.28***	121.65	67.28 ± 0.46***	112.49
	500	59.31 ± 0.28***	​	57.19 ± 0.47***	​
	250	48.84 ± 0.26***	​	47.09 ± 0.43***	​
	125	34.89 ± 0.36***	​	33.64 ± 0.43***	​
	62.5	20.93 ± 0.37***	​	20.18 ± 0.29***	​
Leaf	1,000	65.78 ± 0.22***	129.37	72.1 ± 0.42***	173.99
	500	55.91 ± 0.5***	​	61.29 ± 0.46***	​
	250	46.05 ± 0.54***	​	50.47 ± 0.31***	​
	125	32.89 ± 0.37***	​	36.05 ± 0.47***	​
	62.5	19.73 ± 0.28***	​	21.63 ± 0.33***	​
Stem	1,000	69.15 ± 0.34***	299.68	69.16 ± 0.22***	284.02
	500	58.78 ± 0.36***	​	58.78 ± 0.19***	​
	250	48.41 ± 0.38***	​	48.41 ± 0.27***	​
	125	34.58 ± 0.43***	​	34.58 ± 0.43***	​
	62.5	20.75 ± 0.24***	​	20.75 ± 0.55***	​
Root	1,000	67.44 ± 0.26***	325.48	67.79 ± 0.55***	214.28
	500	57.32 ± 0.29***	​	57.62 ± 0.28***	​
	250	47.2 ± 0.2***	​	47.45 ± 0.31***	​
	125	33.72 ± 0.29***	​	33.89 ± 0.41***	​
	62.5	20.23 ± 0.47***	​	20.34 ± 0.29***	​

*** indicates p < 0.001 versus the positive control.

### 3.3 *In vitro* anti-inflammatory potential

The anti-inflammatory activity of extracts and semi-purified fractions was evaluated by assessing inhibition of COX-2 and 5-LOX enzymes. At 1,000 μg/mL, E-B-3 C exhibited the highest COX-2 inhibition (74.61% ± 0.47%), followed by E-B-2 C (71.83% ± 0.44%) and leaf extract (73.20% ± 0.39%). The root extract showed relatively low inhibition (59.44% ± 0.36%). IC_50_ values ranged from 95.22 to 352.48 μg/mL. Similarly, E-B-1 C demonstrated the highest 5-LOX inhibition (72.94% ± 0.41%) at 1,000 μg/mL, followed by E-B-3 C (70.56% ± 0.33%) and E-B-2 C (68.87% ± 0.35%). The IC_50_ values for 5-LOX inhibition ranged from 104.63 to 341.72 μg/mL (Figures 1, 2). Complete data sets are summarized in Table 1.

## 4 Discussion

The present study systematically evaluated the *in-vitro* inhibitory activity of crude extracts and column semi-purified fractions of *E. brevicornum* against key enzymes involved in bone resorption and inflammation. The results demonstrated concentration-dependent inhibition across all tested targets, with semi-purified fractions generally exhibiting enhanced potency compared to crude extracts. These findings suggest that chromatographic fractionation may enrich bioactive constituents and increase the relative concentration of enzyme-modulatory compounds. Such enhancement following fractionation supports the rationale for phytochemical refinement in identifying active principles (Zhang et al., 2020).

Osteoporosis is characterized by disruption of the dynamic equilibrium between bone formation and bone resorption (Cui et al., 2022). Excessive osteoclastic activity leads to progressive degradation of the organic and inorganic components of bone matrix (Omi and Mishina, 2022; Faulkner et al., 2019). Bone resorption is a coordinated cascade involving osteoclast differentiation, formation of the ruffled border, acidification of the resorption lacuna, and enzymatic degradation of collagen and extracellular matrix (Väänänen and Laitala-Leinonen, 2008). TRAP is considered a hallmark enzyme of mature osteoclasts and is widely used as a biochemical indicator of osteoclast activation (Gnant et al., 2013). Clinically, elevated TRAP activity has been correlated with increased bone turnover and heightened fracture risk (Jørgensen et al., 2021). In the present study, inhibition of TRAP catalytic activity suggests interference with enzymatic processes associated with osteoclast function (Lu et al., 2024). While enzymatic inhibition alone does not confirm suppression of osteoclast differentiation, it provides preliminary evidence that the tested extracts may modulate bone-resorptive activity at the biochemical level.

Cathepsin K represents one of the most critical proteases involved in degradation of type I collagen, the primary structural component of bone matrix. Its expression is highly restricted to osteoclasts, and its enzymatic activity is optimized in the acidic microenvironment created during bone resorption (Xue et al., 2023; Brizuela et al., 2025). Experimental evidence from genetic deficiency models has demonstrated that absence of cathepsin K results in impaired bone resorption and increased bone mass (Brömme et al., 2016). Pharmacological inhibition of Cathepsin K has therefore been explored as a targeted anti-resorptive strategy in osteoporosis therapy (Boonen et al., 2012; Khosla and Hofbauer, 2017). Although certain clinical inhibitors encountered safety concerns, the enzyme remains a validated and biologically relevant therapeutic target. The significant inhibition of cathepsin K activity observed in this study reinforces the anti-resorptive potential of *E. brevicornum*, particularly in semi-purified fractions that exhibited lower IC_50_ values. This pattern implies enrichment of constituents capable of interacting with proteolytic enzymes responsible for collagen degradation.

Matrix metalloproteinases (MMPs), especially MMP-9 and MMP-13, contribute to extracellular matrix remodeling and collagen turnover during bone degradation (Paiva and Granjeiro, 2017; Lei et al., 2013). MMP-9 facilitates osteoclast migration and invasion, whereas MMP-13 participates in collagen turnover and structural remodeling of bone tissue (Guimaraes-Stabili et al., 2020). Increased MMP expression and activity have been reported in pathological bone loss conditions and are associated with compromised bone strength and microarchitecture. The observed inhibition of both MMP-9 and MMP-13 in the present study suggests that *E. brevicornum* extracts may exert broader modulatory effects across multiple components of the resorptive machinery (Chinvattanachot et al., 2024). The parallel inhibition of TRAP, cathepsin K, and MMPs indicates potential multi-enzyme targeting within the bone degradation cascade rather than isolated enzymatic suppression.

Inflammatory processes play an increasingly recognized role in osteoporosis, particularly in postmenopausal and age-related bone loss (Chen et al., 2022). Chronic low-grade inflammation can enhance osteoclastogenesis and amplify bone resorption through upregulation of degradative enzymes and inflammatory mediators (Güler-Yüksel et al., 2018; Imerb et al., 2020). COX-2 and 5-LOX are key enzymes responsible for generating prostaglandins and leukotrienes, which can influence bone turnover dynamics (Raisz et al., 1993; Sankaranarayanan et al., 2024). Elevated COX-2 expression has been associated with increased bone resorption under inflammatory conditions (Pilbeam, 2020). The inhibition of COX-2 and 5-LOX activity observed in this study suggests that *E. brevicornum* may exert anti-inflammatory effects that complement its enzyme-modulatory activity against bone-resorptive targets. The dual inhibition of inflammatory and resorptive enzymes may provide a synergistic advantage in conditions where inflammation and bone loss are interconnected (Iantomasi et al., 2023).

Phytochemical analysis of the crude extract revealed a complex profile dominated by flavonoids and prenylated flavonol glycosides, including icariin, icariside A7, anhydroicaritin, kaempferol, and quercetin (Zhang et al., 2023; Wang et al., 2017). Flavonoids have been extensively studied for their antioxidant, anti-inflammatory, and enzyme-modulatory properties. Prenylation may enhance their lipophilicity and improve interaction with biological membranes and enzymatic binding sites (Lv et al., 2023). Although the present study did not isolate individual active compounds, the enhanced inhibitory activity observed in semi-purified fractions suggests that fractionation may concentrate specific phytochemical classes contributing to enzyme inhibition. Moreover, plant extracts often exert biological effects through additive or synergistic interactions among multiple constituents, which may explain the consistent multi-target inhibition observed across different enzyme systems. This multi-component effect may also explain why the crude extract showed greater apparent potency than pure icariin reported in previous studies. However, direct comparison should be interpreted cautiously because differences in assay conditions and experimental systems may influence the reported potency.

It should be emphasized that enzyme-based *in-vitro* assays provide an initial mechanistic screening platform but do not replicate the full complexity of bone remodeling *in vivo*. Enzymatic inhibition does not directly account for regulatory signaling pathways, cellular differentiation processes, or systemic hormonal influences that govern skeletal metabolism. Nevertheless, such assays offer valuable preliminary evidence of biochemical activity and facilitate identification of promising extracts for further evaluation. Future studies should focus on isolation and structural elucidation of active compounds, assessment in osteoclast differentiation and resorption models, and validation in established *in-vivo* models of osteoporosis to determine translational relevance. The overall study design and principal findings are summarized in Figure 3.

**FIGURE 3:**
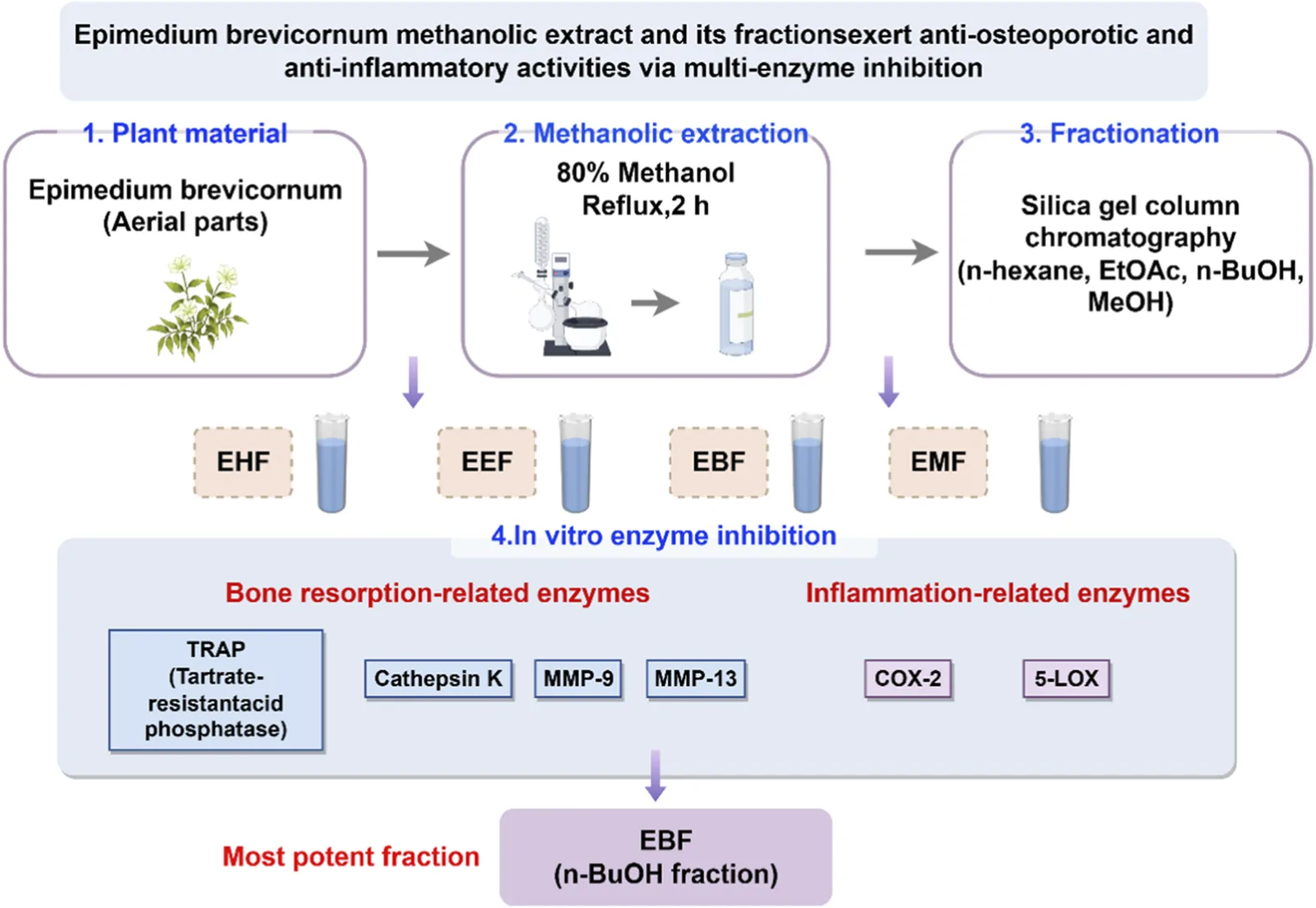
Schematic summary of the overall study design and principal findings. Epimedium brevicornum plant material was subjected to methanolic extraction followed by silica gel column fractionation to obtain four semi-purified fractions (EHF, EEF, EBF, and EMF). The extracts and fractions were evaluated for inhibitory activity against bone resorption-related enzymes (TRAP, cathepsin K, MMP-9, and MMP-13) and inflammation-related enzymes (COX-2 and 5-LOX). Among the tested fractions, EBF (n-BuOH fraction) exhibited the strongest overall inhibitory activity.

## 5 Conclusion

The present study demonstrated that crude extracts and column semi-purified fractions of *E. brevicornum* exhibit concentration-dependent inhibitory activity against key enzymes associated with bone resorption and inflammation. Significant suppression of TRAP, cathepsin K, MMP-9, MMP-13, COX-2, and 5-LOX activity was observed *in vitro*, with semi-purified fractions generally showing enhanced potency compared to crude extracts. These findings suggest that fractionation may enrich bioactive constituents responsible for enzyme modulation. The dual inhibitory activity against bone-resorptive enzymes and inflammatory mediators indicates the potential of *E. brevicornum* as a natural source of compounds relevant to osteoporosis management. However, the present findings are limited to *in-vitro* enzymatic assays. Further studies focusing on isolation and characterization of active constituents, cellular validation, and *in-vivo* investigations are necessary to confirm therapeutic applicability. Overall, this study provides preliminary evidence supporting the enzyme-modulatory and anti-inflammatory potential of *E. brevicornum*, warranting further pharmacological exploration.

## Data Availability

The original contributions presented in the study are included in the article/supplementary material, further inquiries can be directed to the corresponding author.

## References

[B1] Ahirwar V. P. Singhai H. Thakur G. S. Rathee S. Patil U. K. (2026). Mechanistic potentials of natural herbal medicines (NHMs) in promoting bone regeneration: a comprehensive review. Curr. Drug Metab. 27(1), 15-32. 10.2174/0113892002439149251202115833 41716052

[B2] Arena A. C. Leite Kassuya C. A. Konkiewitz E. C. Ziff E. B. (2022). Natural products as sources of new analgesic drugs. Evid. Based Complement. Altern. Med. 2022, 9767292. 10.1155/2022/9767292 35154353 PMC8825241

[B3] Armas L. A. G. Recker R. R. (2012). Pathophysiology of osteoporosis: new mechanistic insights. Endocrinol. Metab. Clin. North Am. 41, 475–486. 10.1016/j.ecl.2012.04.006 22877425

[B4] Boonen S. Rosenberg E. Claessens F. Vanderschueren D. Papapoulos S. (2012). Inhibition of cathepsin K for treatment of osteoporosis. Curr. Osteoporos. Rep. 10, 73–79. 10.1007/s11914-011-0085-9 22228398

[B5] Borrelli J. Lane J. Bukata S. Egol K. A. Takemoto R. Slobogean G. (2014). Atypical femur fractures. J. Orthop. Trauma 28 (Suppl. 1), S36–S42. 10.1097/BOT.0000000000000073 24476604

[B6] Brizuela L. Buchet R. Bougault C. Mebarek S. (2025). Cathepsin K inhibitors as potential drugs for the treatment of osteoarthritis. Int. J. Mol. Sci. 26.(7), 2896. 10.3390/ijms26072896 40243480 PMC11988852

[B7] Brömme D. Panwar P. Turan S. (2016). Cathepsin K osteoporosis trials, pycnodysostosis and mouse deficiency models: commonalities and differences. Expert Opin. Drug Discov. 11, 457–472. 10.1517/17460441.2016.1160884 27001692

[B8] Chen T.-L. Chang K.-H. Su K.-Y. (2022). Effects of biological/targeted therapies on bone mineral density in inflammatory arthritis. Int. J. Mol. Sci. 23.(8):4111. 10.3390/ijms23084111 35456929 PMC9029148

[B9] Chen C. Wang S. Wang N. Zheng Y. Zhou J. Hong M. (2023). Icariin inhibits prostate cancer bone metastasis and destruction via suppressing TAM/CCL5-mediated osteoclastogenesis. Phytomedicine 120, 155076. 10.1016/j.phymed.2023.155076 37716031

[B10] Chen X.-L. Li S.-X. Ge T. Zhang D.-D. Wang H.-F. Wang W. (2024). Epimedium linn: a comprehensive review of phytochemistry, pharmacology, clinical applications and quality control. Chem. Biodivers. 21, e202400846. 10.1002/cbdv.202400846 38801026

[B11] Chinvattanachot G. Rivas D. Duque G. (2024). Mechanisms of muscle cells alterations and regeneration decline during aging. Ageing Res. Rev. 102, 102589. 10.1016/j.arr.2024.102589 39566742

[B12] Cui J. Shibata Y. Zhu T. Zhou J. Zhang J. (2022). Osteocytes in bone aging: advances, challenges, and future perspectives. Ageing Res. Rev. 77, 101608. 10.1016/j.arr.2022.101608 35283289

[B13] Faulkner B. Astleford K. Mansky K. C. (2019). Regulation of osteoclast differentiation and skeletal maintenance by histone deacetylases. Molecules 24. (7), 1355 10.3390/molecules24071355 30959867 PMC6479495

[B14] Gnant M. Baselga J. Rugo H. S. Noguchi S. Burris H. A. Piccart M. (2013). Effect of everolimus on bone marker levels and progressive disease in bone in BOLERO-2. J. Natl. Cancer Inst. 105, 654–663. 10.1093/jnci/djt026 23425564 PMC6430494

[B15] Guimaraes-Stabili M. R. de Medeiros M. C. Rossi D. Camilli A. C. Zanelli C. F. Valentini S. R. (2020). Silencing matrix metalloproteinase-13 (Mmp-13) reduces inflammatory bone resorption associated with LPS-Induced periodontal disease *in vivo* . Clin. Oral Investig. 25, 3161–3172. 10.1007/s00784-020-03644-3 33140162

[B16] Güler-Yüksel M. Hoes J. N. Bultink I. E. M. Lems W. F. (2018). Glucocorticoids, inflammation and bone. Calcif. Tissue Int. 102, 592–606. 10.1007/s00223-017-0335-7 29313071

[B17] Habermann B. Eberhardt C. Feld M. Zichner L. Kurth A. A. (2007). Tartrate-resistant acid phosphatase 5b (TRAP 5b) as a marker of osteoclast activity in the early phase after cementless total hip replacement. Acta Orthop. 78, 221–225. 10.1080/17453670710013717 17464610

[B18] Iantomasi T. Romagnoli C. Palmini G. Donati S. Falsetti I. Miglietta F. (2023). Oxidative stress and inflammation in osteoporosis: molecular mechanisms involved and the relationship with microRNAs. Int. J. Mol. Sci. 24.(4), 3772. 10.3390/ijms24043772 36835184 PMC9963528

[B19] Imerb N. Thonusin C. Chattipakorn N. Chattipakorn S. C. (2020). Aging, obese-insulin resistance, and bone remodeling. Mech. Ageing Dev. 191, 111335. 10.1016/j.mad.2020.111335 32858037

[B20] Jain S. (2024). Prevention and management of denosumab discontinuation rebound fractures. Endocrinol. Metab. Clin. North Am. 53, 559–583 . 10.1016/j.ecl.2024.08.002 39448137

[B21] Jørgensen H. S. Behets G. Viaene L. Bammens B. Claes K. Meijers B. (2021). Diagnostic accuracy of noninvasive bone turnover markers in renal osteodystrophy. Am. J. Kidney Dis. 79, (5), 667-676.e1. 10.1053/j.ajkd.2021.07.027 34710517

[B22] Kanis J. A. McCloskey E. V. Johansson H. Cooper C. Rizzoli R. Reginster J. Y. (2012). European guidance for the diagnosis and management of osteoporosis in postmenopausal women. Osteoporos. Int. 24, 23–57. 10.1007/s00198-012-2074-y 23079689 PMC3587294

[B23] Khosla S. Hofbauer L. C. (2017). Osteoporosis treatment: recent developments and ongoing challenges. Lancet Diabetes Endocrinol. 5, 898–907. 10.1016/S2213-8587(17)30188-2 28689769 PMC5798872

[B24] Lei H. Leong D. Smith L. R. Barton E. R. (2013). Matrix metalloproteinase 13 is a new contributor to skeletal muscle regeneration and critical for myoblast migration. Am. J. Physiol. Cell Physiol. 305, C529–C538. 10.1152/ajpcell.00051.2013 23761625 PMC3761151

[B25] Lu J. Shi X. Fu Q. Han Y. Zhu L. Zhou Z. (2024). New mechanistic understanding of osteoclast differentiation and bone resorption mediated by P2X7 receptors and PI3K-Akt-GSK3β signaling. Cell Mol. Biol. Lett. 29, 100. 10.1186/s11658-024-00614-5 38977961 PMC11232284

[B26] Lv H.-W. Wang Q.-L. Luo M. Zhu M.-D. Liang H.-M. Li W.-J. (2023). Phytochemistry and pharmacology of natural prenylated flavonoids. Arch. Pharm. Res. 46, 207–272. 10.1007/s12272-023-01443-4 37055613 PMC10101826

[B27] Mustafa F. E.-Z. A. Abd-Elhafeez H. H. Taha R. S. Osman A. Almaki M. M. Baker Z. M. (2025). MMP-9 expression in bone cells in the developing femur. Tissue Cell 95, 102862. 10.1016/j.tice.2025.102862 40203681

[B28] Omi M. Mishina Y. (2022). Roles of osteoclasts in alveolar bone remodeling. Genesis 60, e23490. 10.1002/dvg.23490 35757898 PMC9786271

[B29] Paiva K. B. S. Granjeiro J. M. (2017). Matrix metalloproteinases in bone resorption, remodeling, and repair. Prog. Mol. Biol. Transl. Sci. 148, 203–303. 10.1016/bs.pmbts.2017.05.001 28662823

[B30] Pilbeam C. (2020). Prostaglandins and bone. Handb. Exp. Pharmacol. 262, 157–175. 10.1007/164_2019_332 31820176

[B31] Raggatt L. J. Partridge N. C. (2010). Cellular and molecular mechanisms of bone remodeling. J. Biol. Chem. 285, 25103–25108. 10.1074/jbc.R109.041087 20501658 PMC2919071

[B32] Raisz L. G. (2005). Pathogenesis of osteoporosis: concepts, conflicts, and prospects. J. Clin. 115, 3318–3325. 10.1172/JCI27071 16322775 PMC1297264

[B33] Raisz L. G. Pilbeam C. C. Fall P. M. (1993). Prostaglandins: mechanisms of action and regulation of production in bone. Osteoporos. Int. 3 (Suppl. 1), 136–140. 10.1007/BF01621888 8461541

[B34] Ren Z.-Y. Machuca-Gayet I. Domenget C. Buchet R. Wu Y. Jurdic P. (2015). Azanitrile cathepsin K inhibitors: effects on cell toxicity, osteoblast-induced mineralization and osteoclast-mediated bone resorption. PLoS One 10, e0132513. 10.1371/journal.pone.0132513 26168340 PMC4500499

[B35] Sankaranarayanan J. Lee S. C. Kim H. K. Kang J. Y. Kuppa S. S. Seon J. K. (2024). Cinnamaldehyde-mediated suppression of MMP-13, COX-2, and IL-6 through MAPK and NF-κB signaling inhibition in chondrocytes and synoviocytes under inflammatory conditions. Int. J. Mol. Sci. 25, 12914. 10.3390/ijms252312914 39684628 PMC11641634

[B36] Silverstein W. K. Cantor N. Cheung A. M. (2024). Postmenopausal osteoporosis. N. Engl. J. Med. 390, 673–674. 10.1056/NEJMc2314624 38354153

[B37] Ståhle-Bäckdahl M. Sandstedt B. Bruce K. Lindahl A. Jiménez M. G. Vega J. A. (1997). Collagenase-3 (MMP-13) is expressed during human fetal ossification and re-expressed in postnatal bone remodeling and in rheumatoid arthritis. Lab. Invest. 76, 717–728. 9166290

[B38] Udagawa N. Koide M. Nakamura M. Nakamichi Y. Yamashita T. Uehara S. (2020). Osteoclast differentiation by RANKL and OPG signaling pathways. J. Bone Min. Metab. 39, 19–26. 10.1007/s00774-020-01162-6 33079279

[B39] Väänänen H. K. Laitala-Leinonen T. (2008). Osteoclast lineage and function. Arch. Biochem. Biophys. 473, 132–138. 10.1016/j.abb.2008.03.037 18424258

[B40] Walker M. D. Shane E. (2023). Postmenopausal Osteoporosis. N. Engl. J. Med. 389, 1979–1991. 10.1056/NEJMcp2307353 37991856

[B41] Wang Z. Wang D. Yang D. Zhen W. Zhang J. Peng S. (2017). The effect of icariin on bone metabolism and its potential clinical application. Osteoporos. Int. 29, 535–544. 10.1007/s00198-017-4255-1 29110063

[B42] Xue C. Luo H. Wang L. Deng Q. Kui W. Da W. (2023). Aconine attenuates osteoclast-mediated bone resorption and ferroptosis to improve osteoporosis via inhibiting NF-κB signaling. Front. Endocrinol. (Lausanne) 14, 1234563. 10.3389/fendo.2023.1234563 38034017 PMC10682992

[B43] Yang W. Zhu Z. Li L. McVicar A. Gao N. Wang L. (2020). Silencing of Ac45 simultaneously inhibits osteoclast-mediated bone resorption and attenuates dendritic cell-mediated inflammation through impairing acidification and cathepsin K secretion. Infect. Immun. 89, e00436–20. 10.1128/IAI.00436-20 33077625 PMC7927931

[B44] Yong E.-L. Logan S. (2021). Menopausal osteoporosis: screening, prevention and treatment. Singap. Med. J. 62, 159–166. 10.11622/smedj.2021036 33948669 PMC8801823

[B45] Yu B. Wang C.-Y. (2022). Osteoporosis and periodontal diseases - an update on their association and mechanistic links. Periodontol 2000, 89. 10.1111/prd.12422 35244945 PMC9067601

[B46] Zhang L. Song J. Kong L. Yuan T. Li W. Zhang W. (2020). The strategies and techniques of drug discovery from natural products. Pharmacol. Ther. 216, 107686. 10.1016/j.pharmthera.2020.107686 32961262

[B47] Zhang X. Tang B. Wen S. Wang Y. Pan C. Qu L. (2023). Advancements in the biotransformation and biosynthesis of the primary active flavonoids derived from epimedium. Molecules 28, 7173. 10.3390/molecules28207173 37894651 PMC10609448

